# Utilizing a Behavioral Health Bundle to Improve Patient and Clinician Safety for Hospitalized Children

**DOI:** 10.1097/pq9.0000000000000393

**Published:** 2021-03-10

**Authors:** Roger Nicome, Huay-Ying Lo, Sheena Gupta, Adrita Khan, Alice Lee, Wallis Molchen, Hannah Neubauer, Veena Ramgopal, Michelle Lyn, Emily Weber, Joyee Vachani

**Affiliations:** From the Department of Pediatrics at Baylor College of Medicine, Texas Children’s Hospital, Houston, Tex.

## Abstract

Due to limited psychiatric hospital availability, increasing numbers of pediatric patients with behavioral health (BH) needs are hospitalized in medical units in the US Patients and staff are at increased risk for safety events like self-harm or aggression. Our study aimed to decrease safety events by 25% over a year among hospitalized children with BH diagnoses by implementing an intervention bundle.

**Methods:**

A multidisciplinary team developed and implemented a BH intervention bundle that included a BH equipment cart, an electronic medical record tool for BH patient identification/stratification, a de-escalation team, daily operational BH phone call, and staff training with a safety checklist. The primary outcome measure was the number of reported safety events in BH patients. Process measure was “medically avoidable days”, wherein a medically cleared patient remained hospitalized awaiting transfer to inpatient psychiatric units; balance measure was staff perception of the workflow.

**Results:**

Although not statistically significant, we noted a downward trend in safety events per 1,000 patient days from 0.47 preintervention to 0.34 postintervention (28% decrease). Special cause variation was not achieved for BH safety events or medically avoidable days. Although one-third of staff members felt the BH bundle was helpful, many reported it as impeding workflow and expressed ongoing discomfort caring for BH patients.

**Conclusions:**

The implementation of a BH intervention bundle requires significant institutional support and interdisciplinary coordination. Despite additional training, equipment, and staff support, we did not achieve measurable improvements in patient safety and care coordination. Additional studies to measure impact and improve care for this population are needed.

## Abstract 2

Supplemental Digital Content is available in the text.

## INTRODUCTION

Mental health disorders comprise the fourth major diagnostic category for non-newborn pediatric hospitalization and are the most common reason for adolescents’ hospitalization.^1^ Nearly 20% of hospitalized children have either a primary or secondary psychiatric diagnosis.^2^ In the United States, pediatric admissions with a primary psychiatric diagnosis have continued to rise at rates 5 times higher than that of hospitalizations without a psychiatric diagnosis.^3^ Concurrently, there has been a national decline in the availability of inpatient psychiatric beds.^4,5^ This disproportionate model has resulted in psychiatric patients frequently boarding in the emergency department (ED), with up to half being admitted to inpatient units, resulting in longer lengths of stay than patients with primary medical diagnoses.^2,6–9^ This practice leads to an increase in medically avoidable days (MADs), in which the patient is medically clear awaiting inpatient psychiatric placement, inflating length of stay, and cost.^8,10^ Lack of appropriate psychiatric care and standard room setups on inpatient units is a potential safety risk impacting staff and patients.^11^

Early psychiatric consultation has aided in providing behavioral health (BH) evaluation and interventions, identifying opportunities to improve patient safety, determining patient disposition, facilitating transfers/discharges, and collaborating with other members of patients’ medical homes.^12–14^ Early consultation in the inpatient pediatric setting reduced length of stay and total hospital charges.^14^ Some patients showed significant benefit from interventions while boarding and were even diverted from psychiatric hospitalization.^13^ In the pediatric suicidal population, BH safety protocols and safety huddles can significantly decrease serious safety events amongst adolescents hospitalized for medical stabilization after a suicide attempt.^15^

Mental health disorders are the most common cause of hospitalization among adolescents in Texas; consistent with national trends, psychiatric patients boarding in pediatric inpatient units without appropriate psychiatric care experience high lengths of stay and hospital costs and pose safety risks to themselves and others.^16^ This article describes a quality improvement project to implement a bundle of interventions to decrease the rate of safety events by 25% over 1 year in children admitted with a BH diagnosis at a large quaternary children’s hospital.

## METHODS

This project was a quality improvement (QI) study that utilized the Institute of Healthcare Model for Improvement. It was part of a larger comprehensive institutional effort to improve care for BH patients.^17^

### Context

The team implemented this project at a 650-bed free-standing, quaternary pediatric hospital in Houston, Tex., with approximately 32,000 annual hospital admissions. It included BH patients with International Classification of Diseases (ICD)-10 codes F04-F79 or F83-F99 admitted to the inpatient Pediatric Hospital Medicine (PHM) service at the Texas Children’s Hospital medical center campus.

The hospital made the creation of a safe care environment for BH patients an institutional priority in mid-2016. This priority resulted from ongoing growth in this population on the inpatient wards and prolonged hospitalizations due to the limited availability of psychiatric hospitals for children in the community. Stakeholders convened to create a strategy and operationalize an institution-wide approach to improve the quality of care for hospitalized BH patients. Stakeholders included nurses, physicians (PHM, psychiatry, and ED), psychologists, hospital administrators, and staff from facilities and data analytics. They formed 3 subteams to:

- gather data (patient volume, projections, resources, and injuries), formulate a system-wide approach, and
- explore a community partnership strategy after reviewing available evidence in the literature and best practices.

**Fig. 1.**
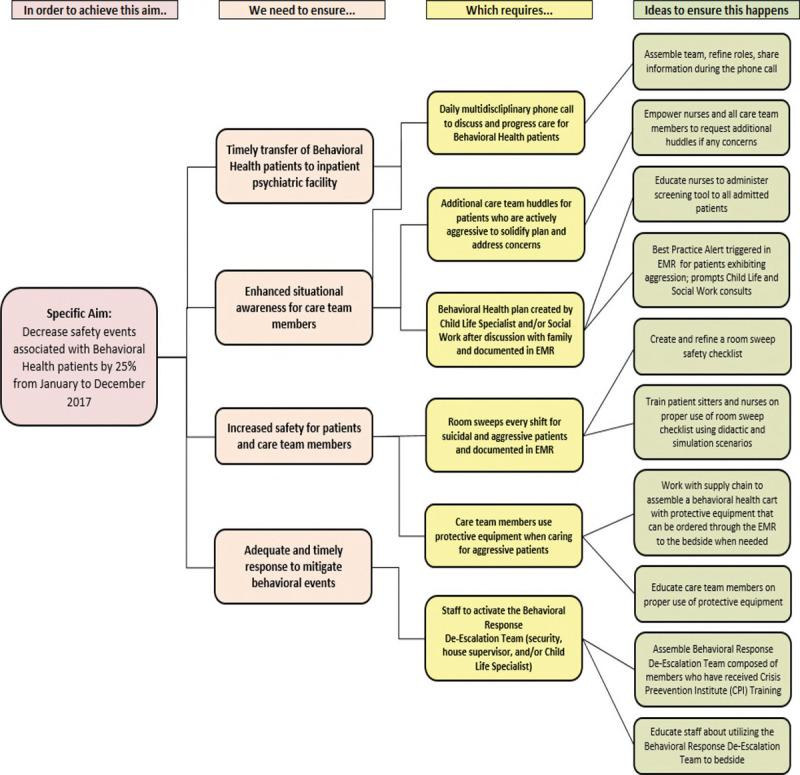
Key driver diagram.

They created a key driver diagram (Fig. 1) and implemented sequential interventions over a year (Table 1), anticipating iterative improvement.

**Table 1.** Timeline

PDSA Cycle	Month Initiated (2017)	Details
1	January	Personal protective equipment cart, admission screening tool for aggression in EPIC
2	February	De-escalation team, suicide screening in the ED
3	April	Daily BH phone call
4	July and August	Nursing room safety sweep process implemented system-wide, patient sitter simulation training
5	September	Policy for scope of services and transfer criteria defined for all campuses

### Plan-Do-Study-Act Cycle 1

The first Plan-Do-Study-Act (PDSA) cycle involved creating a personal protective equipment cart for use with an aggressive patient. Central supply stocked the cart, and providers ordered it through the electronic medical record (EMR). It contained Kevlar sleeves, face-masks with shields, isolation gowns, and coated gloves. Users provided feedback on the contents of the cart for iterative improvement. The team internally developed an EMR-based tool to screen admitted patients for aggression (**see Appendix A,** which describes behavioral health team tools, **Supplemental Digital Content 1,** http://links.lww.com/PQ9/A243) by assigning points for specific behaviors. Screened patients exhibiting a propensity toward aggression received consults from a social worker and child life specialist. They worked with the family to create a support plan, including aggressive behavior triggers and support strategies for the patient, family, and care team. This plan was uploaded to the EMR and remained accessible to the care team during the inpatient admission and subsequent encounters.

### PDSA Cycle 2

A de-escalation team comprising security, child life, social work, and the on-call nursing house supervisor, received the Crisis Prevention Institute training to use nonviolent measures to de-escalate aggressive patients.^18^ The full team could be rapidly summoned to the bedside between 8 am and 5 pm on weekdays, with a limited team consisting of security and nursing house supervisors available after-hours and on weekends. Physicians were available to order restraints per regulatory guidelines if initial de-escalation strategies were unsuccessful. During this timeframe, the ED implemented suicide screening for all patients 11 years or older, allowing for early initiation of suicide precautions before admission.

### PDSA Cycle 3

The next intervention implemented was a daily morning phone call with representatives from social work, care management, nursing, psychology, PHM, psychiatry, security, child life, patient safety/quality, and family advocacy to discuss patients with active BH concerns. This call was under 30 minutes and actively facilitated by the psychiatrist to discuss hospitalized BH patients with ongoing needs beyond medical clearance to ensure the allocation of resources to progress care. The call was iteratively improved to address concerns and increase situational awareness so that the medical teams could successfully implement their daily care plan. If necessary, patients with extreme aggression or multiple aggressive episodes could incur additional care-team huddles later in the day with security present to provide updates and ensure adherence with safety protocols. Psychiatry maintained and shared an updated patient list with the team daily with demographic information, medical and psychiatric diagnoses, psychiatric treatment with contingencies, disposition, and an indication of risk (elopement, self-harm, and harm to others).

### PDSA Cycle 4

The next intervention focused on providing mandatory training, including the Crisis Prevention Institute training for frontline nursing and sitter staff. A total of 515 staff, including nurses, patient care assistants (PCAs), and patient sitters, received training. Participants reviewed an online module then attended a 1- to 2-hour workshop with an interactive lecture followed by skills stations with role play. Participants practiced EMR documentation in simulated case scenarios using a standardized safety checklist to build competence in detecting and mitigating potential safety breaches (**see Appendix A,** which discribes behavioral health team tools, **Supplemental Digital Content 1,** http://links.lww.com/PQ9/A243). The team iteratively refined the checklist to optimize the safety of patients and care team members. The checklist was completed once per 12-hour shift by sitters and nurses; the sitters documented in the EMR. Physicians could make exceptions to the checklist only after consultation between the pediatricians and psychiatrists.

### PDSA Cycle 5

In addition to the safety checklist, the team also standardized the scope of services managed at the hospital system’s community sites. The Texas Children’s system consists of a medical center campus and 2 community sites. There is no onsite psychiatric physician presence at the community sites. However, psychiatrists provide limited telephonic consultation services. Stakeholders helped define which patients could be safely managed at the community sites and which should transfer to the medical center site for psychiatric consultation (including patients who had a comorbid intellectual disability, required daily medication management, or anticipated post-hospital disposition challenges). Transfers to the medical center campus from the community sites accounted for about one patient per month.

### Study of the Interventions and Measures

The team implemented serial interventions between January and October 2017 and obtained data on preintervention (January to December 2016) and postintervention (January to December 2017) safety event rates and MADs from electronic databases maintained by our institution’s hospital quality and safety teams. A report of BH patients admitted during the study period was generated in our EMR Epic (Epic Systems Corporation, Verona, Wis.), which included children admitted with ICD-10 codes for BH issues.

### Outcome Measures

The primary outcome measure was the number of “safety scoops” filed for patients with BH diagnoses. Any hospital staff can file these safety event reports online using the hospital intranet, and they can choose to self-identify or remain anonymous. Safety scoops have existed since 1996 and are strongly endorsed by institutional quality and safety leaders as a means to investigate perceived safety events. Users can categorize safety scoops by type and enter a free text description of the safety event.

Study authors obtained a list of safety scoops filed for the BH patient cohort. Safety scoop rates were tracked per 1,000 patient days to account for any seasonal variation in the number of related psychiatric hospitalizations. Secondary outcome measures were obtained from a staff perception survey of registered nurses and PCAs to assess whether the bundle was helpful and if staff members felt safer when caring for BH patients. The study team members designed the survey to preserve respondent anonymity and obtained approval from nursing leadership before administering the survey. It was distributed once in electronic and paper formats to staff on 3 PHM primary hospital units. We collected responses over 3 weeks.

### Process Measure

The process measure was MADs—the number of excess days a medically cleared patient remains hospitalized due to care delivery- or discharge-related barriers. The care coordination department tracked this number and provided MADs per 1,000 patient days with “psychiatric” as the reason for continued admission.

We chose MADs as a process measure and potential surrogate marker for overall improved care coordination because it made MAD reduction an institutional priority. We hoped that the implementation of daily BH huddles and dedicated care management assistance to streamline care would expedite transfers to inpatient psychiatric facilities once patients were medically clear.

Additional process measures included the number of times the BH cart was ordered and the pages to the de-escalation team per month.

### Balance Measure

Staff perception surveys assessing whether the new interventions impeded workflow served as our primary balance measure. We also acknowledge the financial costs of our BH intervention bundle, including the cost for providing the Crisis Prevention Institute training for staff.

### Statistical Analysis

The team created statistical process control (SPC) charts in Excel QI Macros (KnowWare International, Denver, Colo.) to track outcome and process measures over time, and applied established rules for determining special cause variation. Pearson Chi-Square testing compared categorical pre- and post-intervention metrics; statistical significance was defined as *P* value <0.05.

### Ethical Considerations

The local Institutional Review Board approved this study.

## RESULTS

### Population

When comparing pre- and post-intervention populations, there were no significant differences for age, gender, or race (Table 2). Compared to the postintervention period, the preintervention period had more non-Hispanic patients and more private or commercially insured patients with psychiatric diagnosis codes; there was no difference in self-pay patient percentage.

**Table 2.** Comparison of Patient Demographics Preintervention and Postintervention

	Preintervention, 2016 N = 2894 (48.6%), N (%)	Postintervention, 2017 N = 3064 (51.4%), N (%)	P*
Age (y)			
0–4	610 (21.1%)	578 (18.9%)	0.10
5–10	749 (25.9%)	822 (26.8%)	
>10	1535 (53.0%)	1664 (54.3%)	
Gender			
Female	1329 (45.9%)	1373 (44.8%)	0.39
Male	1565 (54.1%)	1691 (55.2%)	
Race			
Caucasian	2189 (75.6%)	2319 (75.7%)	0.74
African American	530 (18.3%)	573 (18.7%)	
Other	175 (6.0%)	172 (5.6%)	
Ethnicity			
Non-Hispanic	1731 (59.8%)	1764 (57.6%)	0.03
Hispanic	1105 (38.2%)	1267 (41.4%)	
Insurance status			
Private	1126 (38.9%)	1072 (35.0%)	0.007
Public/government	1606 (55.5%)	1810 (59.1%)	
Other/self-pay	162 (5.6%)	182 (5.9%)	

*The *P* value was calculated using Pearson Chi-Square testing.

### Outcome Measures

**Fig. 2.**
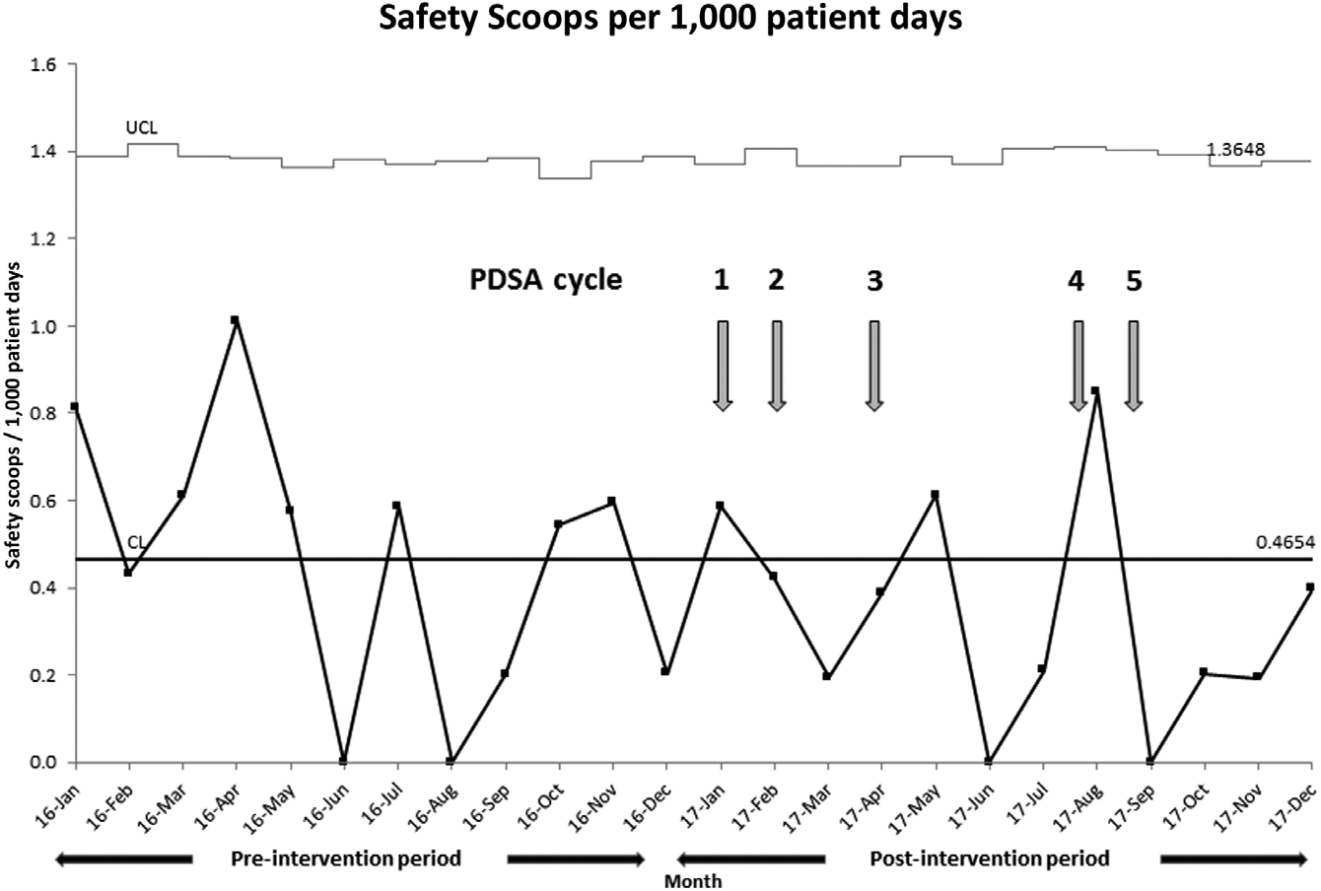
U-chart for Behavioral Health Safety Scoops filed per 1,000 patient days. The centerline and control limits were calculated based on preintervention data (January to December 2016) and extended because special cause variation was not achieved.

The total number of safety scoops and mean/median safety scoops per month decreased in our post-intervention period (Table 3). The number of safety scoops per 1,000 patient days decreased from 0.47 in the preintervention period to 0.34 in the postintervention period. However, this was not statistically significant through either nonparametric testing (*p* value = 0.27) or via SPC chart rules for special cause variation (Fig. 2).

**Table 3.** Comparison of the Number of Safety Scoops and MADs in Preintervention and Postintervention Time Periods

	Preintervention (2016)	Postintervention (2017)
Total patient days	60,166	59,456
Total safety scoops	28	20
Median safety scoops per month	3	1.5
Mean safety scoops per 1,000 patient days	0.47	0.34
Total MADs	122	172
Median MADs per month	8.5	11.5
Mean MADs per 1,000 patient days	2.1	2.9

The staff perception survey of nurses and PCAs in the postintervention period had a response rate of 36% (53/149). Results revealed that 38% (20/53) of respondents felt safe caring for BH patients, whereas 40% (21/53) did not; 23% (12/53) were neutral. One-third (16/48) of respondents felt that the BH intervention bundle made them feel safer, although 38% disagreed. Staff had mixed responses on whether the BH interventions were helpful (33% agree, 17% disagree, and 50% neutral). Responses to questions regarding feedback on the BH intervention bundle and how to improve care for BH patients generally demonstrated discomfort with caring for this patient population, and desire for cohorting patients on a specialized unit with appropriate physical modifications and additional staff training.

### Process Measure

**Fig. 3.**
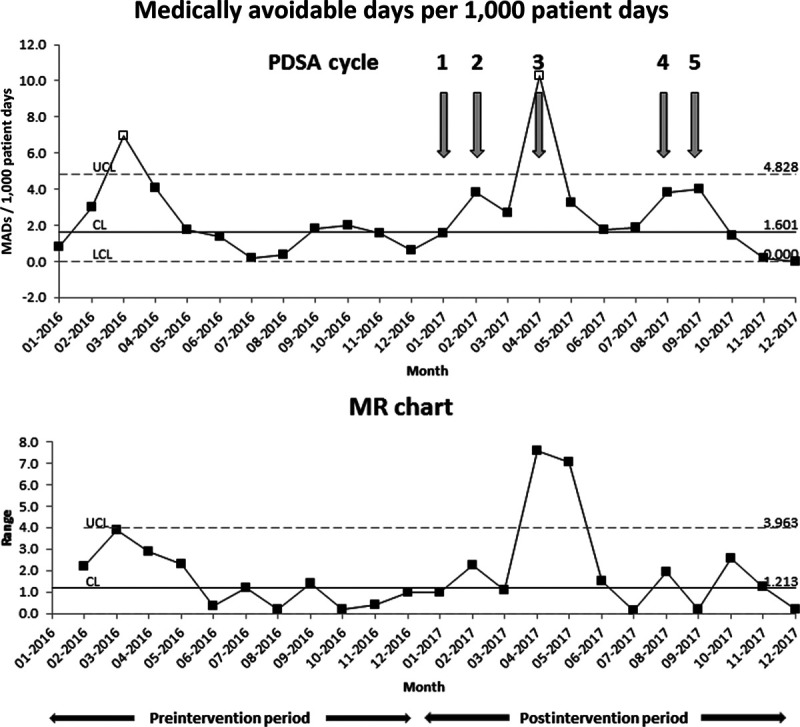
I-MR chart for MADs per 1,000 patient days. The centerline and control limits were calculated based on preintervention data (January to December 2016) and extended. Two months (March 2016 and April 2017) were excluded due to a few patients with significantly prolonged lengths of stay and a high number of medically avoidable days, which skewed the data for those months.

The total, mean, and median number of MADs increased in our post-intervention compared to pre-intervention (Table 3, *P* < 0.01). However, when plotted on an SPC chart (Fig. 3), the mean number of MADs per 1,000 patient days appeared relatively stable. One month each in the preintervention (March 2016) and postintervention (April 2017) periods were excluded from the I-MR chart due to being outliers associated with an unusually high number of patients waiting for psychiatric beds. Several of them had very prolonged MADs, which skewed the numbers for those months.

The BH cart containing safety personal protective equipment was ordered 17 times during the postimplementation period, or approximately 1.4 times per month. Staff activated the de-escalation team 16 times between April and October.

### Balance Measure

Nearly two-thirds of respondents indicated that interventions impeded their workflow (64%, n = 34/53), while 30% were neutral.

## DISCUSSION

### Summary and Interpretation

Our bundle of interventions included creating a BH safety cart and an EMR-based screening tool, a de-escalation team, a daily multidisciplinary phone call, staff training targeting de-escalation, room safety checklists (**see Appendix B, Supplemental Digital Content 2,** http://links.lww.com/PQ9/A244), and standardization of main and community hospital site capabilities. These sequential interventions were, in large part, important because they aligned with institutional initiatives and involved numerous critical stakeholders throughout the process.

To date, there has been one study investigating serious safety events with adolescents hospitalized after suicide attempts.^15^ This study is the first QI initiative focused on safety events in patients hospitalized with any BH diagnosis. Although our results were not statistically significant, we felt that any improvement in patient or staff safety is meaningful. Still, our inability to significantly decrease safety events despite a thoughtful, multidisciplinary approach highlights the difficulty of caring for these patients. Several components of our bundle, including the BH safety cart and de-escalation team, were utilized by staff members caring for BH patients, but safety events and MADs were unchanged. Many of these diagnoses can manifest as unpredictable, volatile behavior, so inevitably safety issues arise. Although our goal of reducing safety events in these patients is desirable, perhaps they will continue to occur, so hospitals should strive to provide the safest possible environment to mitigate them.

The authors hypothesized that the interventions would decrease MADs due to enhanced care coordination and communication among team members caring for BH patients. Although aggregate total and mean MADs were higher in the postintervention period than in the preintervention period, no special cause variation was detected on the SPC chart. We suspect that this is because enhanced care coordination among our team has no impact on inpatient psychiatric bed availability for children, which is a significant driver for MADs. We noted outlier months in both the preintervention and postintervention periods during which MADs were significantly higher than usual, due to a higher number of patients awaiting inpatient psychiatric beds. Several of them had protracted waits over ten days. The postintervention period also contained a higher percentage of patients with public insurance, which sometimes limits placement options; this may also have contributed to increased MADs.

Staff survey responses indicated varying levels of comfort in caring for these patients. About one-third reported that the BH interventions helped them feel safer. A majority of staff reported that the BH intervention bundle increased their work—likely due to the room sweeps, screenings, and checklists implemented to create a safe environment for BH patients.

Although other hospitals may not implement the entire bundle, individual components of the bundle may help facilitate care coordination and improved safety for hospitalized BH patients. Additional staff training and other initiatives to improve care for these patients are critical to ensuring patient and staff safety. Further studies are needed to assess the impact of various interventions on improving the care provided for this growing patient population.

Our hospital is monitoring the ongoing sustainability of these results. There has been an institutional culture shift with increased awareness of bundle components. Hospital leadership support of these initiatives is crucial to the continued success of this program. The next steps include additional staff training to improve staff comfort and safety in caring for these patients and creating dual-purpose rooms on one unit to facilitate cohorting staff and resources unique to this patient population.

### Limitations

There were several limitations to this study. Safety scoop reporting is a voluntary, self-reporting process, so it may not accurately capture the actual number of safety events. Our baseline rate of safety events is low, so this may have limited our ability to detect a statistically significant difference. The sequential roll-out of the interventions also made it difficult to assess if a single bundle component was most impactful. Perhaps looking at a more extended intervention period or having available data for additional outcome measures may have demonstrated more impact on safety events. Despite these limitations, qualitative data collected were generally supportive of improved patient safety.

Our demographic analysis also indicated a difference in insurance status and ethnicity between preintervention and postintervention patients. The presence of more publicly funded patients and/or patient comorbidities such as autism may have impacted MADs. These patients have fewer inpatient psychiatric care options and, once medically cleared, can remain hospitalized for more extended periods as they await transfer. Unfortunately, our tracking systems for safety scoops and MADs do not correlate with individual patient demographic data, so additional subgroup analysis for these populations could not be performed. We are continuing data collection to assess the long term impact of these interventions.

A limitation of the perception survey was the inability to collect preintervention surveys as a comparison. Culture change takes time; another fundamental limitation was staff uptake and sustainability of some of the proposed interventions for this relatively small but potentially disruptive patient population. Furthermore, although we performed a staff survey, we did not perform parental and family surveys to assess their perception of our interventions, and whether they felt the additional security measures and training led to increased safety for their child.

## CONCLUSIONS

This study describes implementing a bundle of BH interventions targeted at improving a safe environment, staff training, availability of appropriate equipment and trained personnel, and care coordination. The discussion above is valuable for others thinking of strategies to reduce BH-related events. Additional efforts and research studies are needed to measure impact and ensure patient and staff safety when caring for this patient population.

## DISCLOSURE

The authors have no financial interest to declare in relation to the content of this article.

## ACKNOWLEDGMENTS

The authors thank Joan Shook, MD, MBA, Laurel Williams, DO, Texas Children’s Hospital for the assistance with the study.

## Supplementary Material


