# Supplementary material for: Utilizing a Behavioral Health Bundle to Improve Patient and Clinician Safety for Hospitalized Children
Source: Pediatr Qual Saf. 2021 Mar 10;6(2):e393. doi: 10.1097/pq9.0000000000000393 (PMC7952108; doi:10.1097/pq9.0000000000000393)

# APPENDIX A: Behavioral Health Team Tools

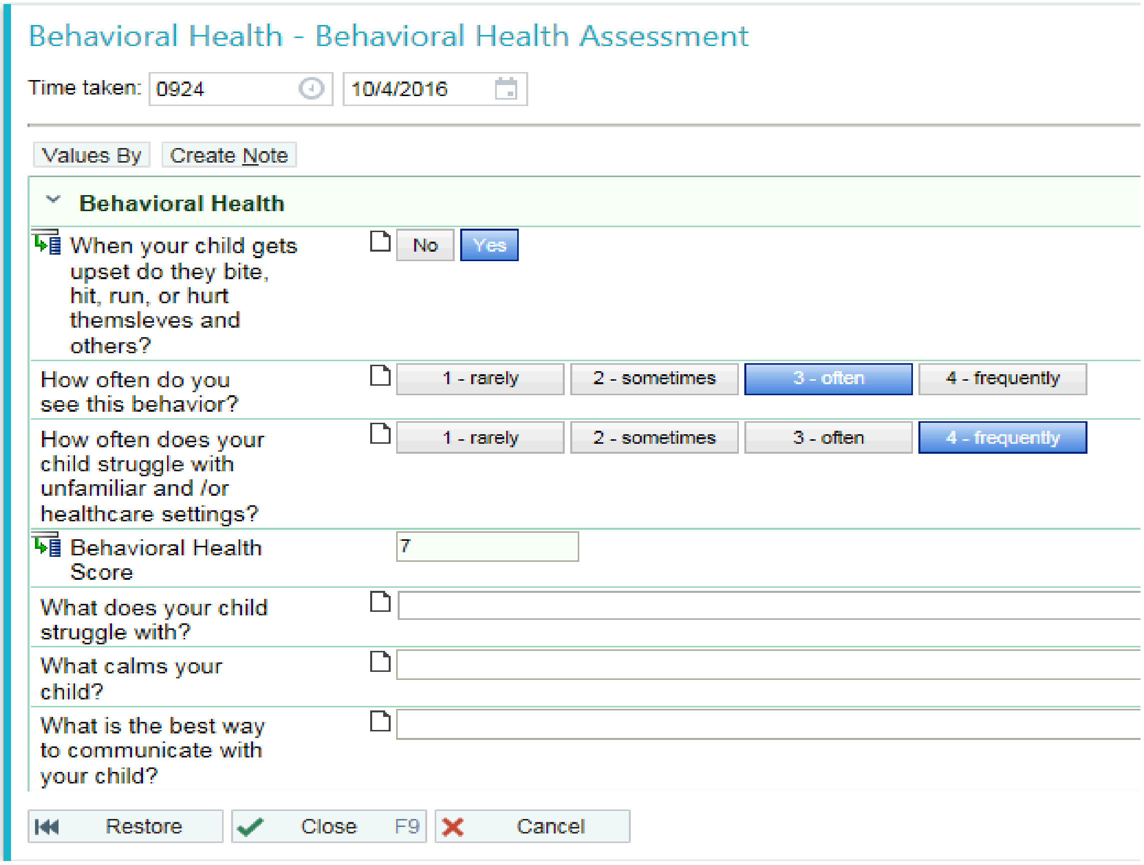

Behavioral Health - Behavioral Health Assessment

Time taken: 0924 10/4/2016

Values By | Create Note

**Behavioral Health**

| | |
|---|---|
| When your child gets upset do they bite, hit, run, or hurt themsleves and others? | No / **Yes** |
| How often do you see this behavior? | 1 - rarely / 2 - sometimes / **3 - often** / 4 - frequently |
| How often does your child struggle with unfamiliar and /or healthcare settings? | 1 - rarely / 2 - sometimes / 3 - often / **4 - frequently** |
| Behavioral Health Score | 7 |
| What does your child struggle with? | |
| What calms your child? | |
| What is the best way to communicate with your child? | |

Restore | Close F9 | Cancel

## Cart Supplies

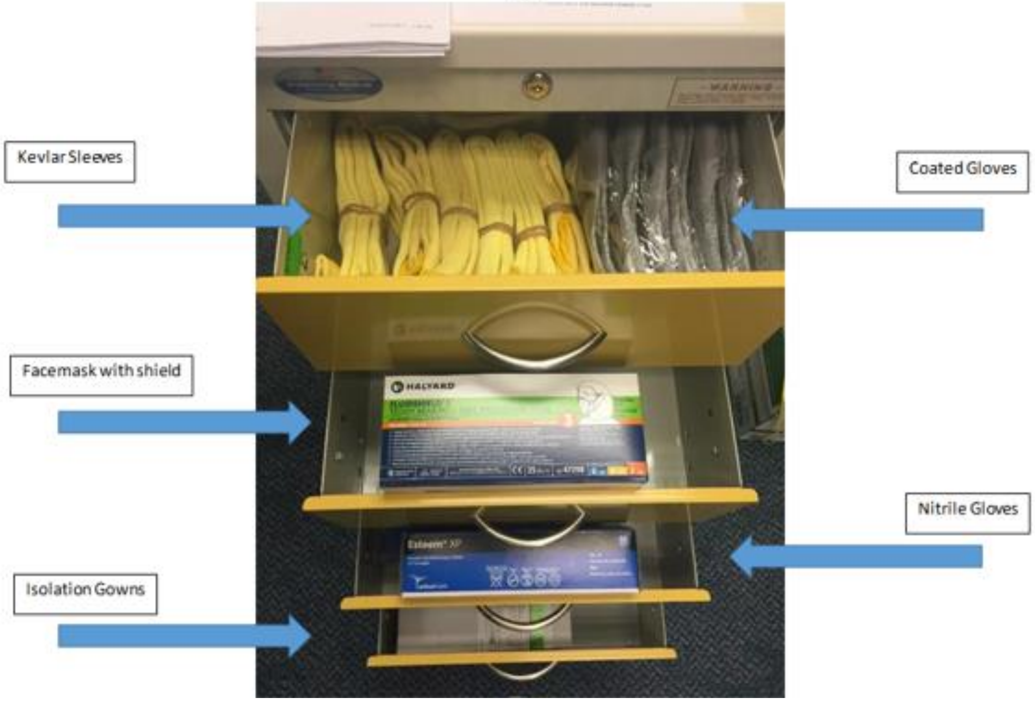

Supplement: Supplementary file 1 [file pqs-6-e393-s001.pdf]
