# Supplementary material for: Utilizing a Behavioral Health Bundle to Improve Patient and Clinician Safety for Hospitalized Children
Source: Pediatr Qual Saf. 2021 Mar 10;6(2):e393. doi: 10.1097/pq9.0000000000000393 (PMC7952108; doi:10.1097/pq9.0000000000000393)
Supplement: Supplementary file 2 [file pqs-6-e393-s002.pdf]

Room Sweep Checklist

Suicidal and Aggressive Patients Requiring Patient Sitters

General Guidelines

Admission Sweep: yes no

Patient Sitter & Other Staff:

- ☐ Patient - observed directly at all times by patient sitter (including inside the bathroom)
- ☐ Staff – remove personal items that could be utilized to harm staff (stethoscope, badge lanyard, ties, etc.)

Room Items:

- ☐ Corded phone removed from room
- ☐ Oxygen flowmeter, Christmas tree, ambu-bag, suction regulator stored safely away from patient access
- ☐ Monitor cables removed; secured (if in use)
- ☐ Otoscope/Ophthalmoscope and cables removed from room
- ☐ Unsecure wall items removed from room (plastic hooks, etc.)

Patient Related Items:

- ☐ **Patient's belongings inspected**, in family's presence, in order to prevent access to unsafe items
- ☐ **Family belongings inspected** for items such as meds, fingernail files, knives, etc.
- ☐ Check devices before and after use for removable parts. Remove from room for any safety concerns
- ☐ Inspect any items with batteries to ensure they are properly secured
- ☐ Plastic utensils only allowed with meals- must count plastic utensils before and after each meal
- ☐ No can drinks – must be poured into a cup

Patient not to have any of the following items in their possession:

- ❑ **Electronic devices (phone, IPOD, games, etc.) are only permitted with a provider order**
- ❑ Medications
- ❑ Glass objects / Sharp metal objects
- ❑ Hair grooming utensils, hair ties removed
- ❑ Jewelry
- ❑ Pencils / Fountain or ball point pens
- ❑ Scissors
- ❑ Matches / Lighters
- ❑ Ropes
- ❑ Razors
- ❑ Hand held mirrors
- ❑ Electrical cords or appliances
- ❑ Removable items on wall or door (example: plastic hooks)

Additional Precautions - Suicidal Patient Only:

- ❑ **Electronic devices (phone, IPOD, games, etc.) are not permitted**
- ❑ Patient wearing doubled hospital gowns (gowns and underwear are the only clothing allowed)
- ❑ Shoelaces / belts / hoodie strings
- ❑ Plastic trash/linen bags – Note: trash and linen stored outside room
- ❑ Shower curtain – Note: contact facilities for proper storing
- ❑ Any packages or belongings brought to patient during admission inspected by nursing staff
- ❑ Visitation restricted to parents or primary caregivers unless specifically ordered by the attending physician

Note: Must have an MD order for any exceptions.

PATIENT LABEL

Completed by: ______________________________

Date/Time: ______________________________

Scanned by: ______________________________
